# Metaproteomic Dataset on Semi‐Diurnal Variability of the Bacterioplankton Communities During a Spring Phytoplankton Bloom in the North Sea

**DOI:** 10.1002/pmic.70001

**Published:** 2025-06-23

**Authors:** Vaikhari Kale, Jürgen Bartel, Daniel Bartosik, Philip Berhard Lude, Chandni Sidhu, Hanno Teeling, Rudolf Amann, Thomas Schweder, Dörte Becher, Anke Trautwein‐Schult

**Affiliations:** ^1^ Institute of Microbiology University of Greifswald Greifswald Germany; ^2^ Institute of Pharmacy University of Greifswald Greifswald Germany; ^3^ Institute of Marine Biotechnology e.V. Greifswald Germany; ^4^ Max Planck Institute for Marine Microbiology Bremen Germany

**Keywords:** *Aurantivirga*, day‐night variability, metaproteomics, phytoplankton bloom, *Candidatus* Prosiliicoccus

## Abstract

Phytoplankton blooms create a substrate‐rich environment that supports the growth of bacterial planktonic heterotrophs. Previously, we studied the dynamics of such bacterioplankton at a long‐term ecological research site near the coast of Helgoland Island (North Sea) once a day. Here, we present a novel dataset (available under the PRIDE‐ID: PXD055396) indicating significant differences at the protein level in a semi‐diurnal analysis. Using metaproteomics, we studied changes in the free‐living (0.2–3 µm) bacterial community that occurred between early (7 am) and late (9 pm) sampling over 3 days. The results highlight the sensitivity, robustness, and reproducibility of mass spectrometry‐based metaproteomic analyses to assess changes in the activities of the bacterioplankton communities. Taxonomic analyses revealed significant changes in the abundance of 65 bacterial genera. Particularly, proteins from the flavobacterial genera *Candidatus* Prosiliicoccus and *Aurantivirga* were significantly more abundant in the late samples. This comprehensive dataset highlights semi‐diurnal changes in bacterial community composition and metabolic activity during a phytoplankton bloom that would have remained undetected with a once‐per‐day sampling approach.

**Abbreviations**
CAZymes: carbohydrate‐active enzymes
LTER: long‐term ecological research
MAGs: metagenome‐assembled genomes
PCoA: principal coordinate analysis
*R* ^2^
: Pearson correlation coefficient

1

Just as other meta‐omics technologies, microbial metaproteomics expands the application of proteomics from pure cultures and simple model systems to complex biological systems consisting of entire microbial communities and possibly host cells [1]. However, conducting metaproteomic studies is often challenging due to sample diversity and complexity [2, 3, 4, 5]. Over the past decade, advancements in the preparation of complex samples and innovative methods of data acquisition and analysis have allowed for a more widespread and routine use of metaproteomics. In recent years, numerous metaproteome studies have provided unique insights into the activity of microbial communities in various environments, such as the oceans [6], the human gut [7, 8, 9], soil [10, 11], wastewater treatment plants [12, 13], and even extreme habitats [14] like high‐CO_2_ aquifers [15]. Studies focusing on the microbial nutrient transport in the South Atlantic Ocean [16], biogeochemical processes within the oxygen minimum zones at a seasonally stratified fjord [17], or the dynamics of the carbon flux in the marine ecosystems [18, 19] showed the potential and challenges of ocean metaproteome studies [6].

Marine microbiomes have increasingly garnered attention due to their impact on global biogeochemical cycles. Marine phytoplankton blooms can trigger large‐scale bacterioplankton blooms, which, though short‐lived, play a crucial role in marine carbon cycling [20, 21, 22]. The blooming and subsequent decay of algae releases substantial amounts of organic matter, including large amounts of various algal polysaccharides. Distinct clades of heterotrophic planktonic bacteria benefit from these substrates during bloom events. Bacterial polysaccharide utilization specialists degrade algal polysaccharides via specific carbohydrate‐active enzymes (CAZymes) [20, 23, 24]. Due to their usually high substrate specificity, a diverse array of such CAZymes is required for the complete breakdown and utilization of each distinct algal polysaccharide [25, 26, 27, 28]. In conjunction with CAZymes, specific types of TonB‐dependent transporter proteins allow these bacteria to bind and transport longer oligosaccharides into the periplasm [23, 29, 30].

During phytoplankton blooms, dynamic changes in environmental conditions, nutrient availability, and substrate supply, as well as in interactions among organisms (animals, zooplankton, phytoplankton, bacterioplankton, and viruses), result in highly dynamic marine microbiome composition. Additionally, phototrophic organisms exhibit a specific temporal relationship between transcriptome, proteome, and metabolome, due to the presence of light‐dark cycles [31, 32], adding another level of complexity in these dynamic systems. For instance, in a controlled light‐dark in vitro study of the phototrophic marine cyanobacterium *Prochlorococcus* MED4, significant variabilities were detected in the relationship between mRNA and protein abundances, with more pronounced oscillations in transcript abundances and later peaks in protein abundances [31]. Several in situ studies on diel oscillation have been conducted in marine ecosystems with surface water temperatures above 20°C, such as the North Pacific Subtropical Gyre, the Daya Bay in the South Chinese Sea, and the North Atlantic Ocean near New Jersey [33, 34, 35], but lacking for temperate marine ecosystems. Some studies confirmed diel oscillations of around half of all *Prochlorococcus* transcripts [36], and 70% of the targeted metabolites [37]. These studies highlight that different effects can be observed on the transcript, protein, and metabolite levels.

To monitor changes in plankton composition, a long‐term ecological research (LTER) site was established off the coast of Helgoland Island in the rather cold (about 10°C water surface temperature during springtime) southern North Sea (“Kabeltonne”, 54°11.3′N, 7°54.0′E, DEIMS.iD: https://deims.org/1e96ef9b‐0915‐4661‐849f‐b3a72f5aa9b1). For this LTER, phytoplankton data has been systematically collected since 1962, and zooplankton data since 1975, together with accompanying physicochemical data [38, 39]. Regular sampling of bacterioplankton at the Helgoland Roads LTER site has been conducted since 2009 and analyzed using a combination of methods, including state‐of‐the‐art metaproteomics [20, 22, 40, 41, 42, 43, 44]. These long‐term sampling campaigns revealed that the complex bacterial communities that thrive during and after phytoplankton blooms are dominated by annually recurrent clades [40, 45] with substantial changes in the relative abundances, sometimes within a day or two. It was recently demonstrated that, especially during spring blooms with little top‐down (predator) control, the composition of this bacterial community is significantly shaped by the availability of dissolved algal glycans, as well as by bacterial glucans that living bacteria recycle from bacterial necromass [43]. In 2020, we sampled at the Helgoland Roads LTER site between 2nd March and 26th May. Previously, we published data on total bacterial cell counts and cell counts for specific clades, chlorophyll *a*, wind direction data, physicochemical and phytoplankton, glucan and saccharide measurements, as well as metagenomes, metatranscriptomes, and additionally for 15 sampling time points (7 am), metaproteomics data [42, 43, 44].

So far, diurnal changes have been out of the scope of the long‐term studies. Here, we applied a metaproteomics workflow in a temperate marine ecosystem (Table S1) to investigate short‐term changes in the free‐living (0.2–3 µm) bacterial community. We used samples collected at semi‐diurnal intervals at 7 am (“early”) and 9 pm (“late”) for 3 days (5th, 6th, and 7th May 2020) and compared them with samples taken before (29th April, 7 am) and afterwards (11th May, 7 am). The early samples were taken between 67 (29th April, 7 am) to 91 min (11th May, 7 am) after sunrise, and the late samples were taken 10 min (5th May, 9 pm) to 14 min (7th May, 9 pm) before sunset (Table S1). Here, we present brief first insights into short‐term changes in the community compositions and activities of the free‐living bacterioplankton communities that were sampled with different time intervals of 10 (between 9 pm and 7 am) and 14 h (between 7 am and 9 pm). Therefore, seawater samples were collected at 1 m depth and sequentially filtered through 142 mm polycarbonate membrane filters (Millipore, Schwalbach, Germany) with decreasing pore sizes (10, 3, and 0.2 µm) as previously described [43] and in the Supporting Information. The proteins of the free‐living bacterial community were extracted from one‐eighth of a 0.2 µm filter by adding lysis buffer, heating, and sonication treatment in triplicate. After centrifugation (1 min, 4°C, 5000 × *g*), the proteins in the supernatant were precipitated by adding pre‐cooled trichloroacetic acid (20% [v/v]), pelleted via centrifugation (1 h, 4°C, 12,000 × *g*), washed three times with pre‐cooled acetone and dried. The remaining protein pellet was resuspended in 2× Laemmli SDS sample loading buffer (4% SDS, 20% glycerol, 10% 2‐mercaptoethanol, 0.002% bromophenol blue in 0.125 M Tris‐HCl [pH 6.8]) and incubated for 5 min at 95°C before separation via SDS‐PAGE (Criterion TG 4%–20% Precast Midi Gel, BIO‐RAD Laboratories, Inc., Hercules, CA, USA). Afterwards, the gel was fixated (40% ethanol [v/v], 10% acetic acid [v/v]), and stained with Coomassie [46], before each gel line was cut into 20 equally sized pieces. The gel pieces were cut into smaller pieces, destained three times with washing buffer (200 mM ammonium bicarbonate in 30% ACN [v/v]), and dehydrated as described by Bonn et al. [47] before the proteins were in‐gel reduced and alkylated. After washing, dehydrating, and drying of the gel pieces, the proteins were tryptically digested (2 µg/mL trypsin, Promega, Fitchburg, WI, USA), incubated for 15 h at 37°C and eluted first with 120 µL solvent A (0.1% acetic acid [v/v]) and second with 100 µL 30% ACN (v/v) for 15 min in an ultrasonication bath (SONOREX SUPER RK 102 H, Bandelin, Berlin, Germany). The pooled eluates were desalted via ZipTip C18 (Merck Millipore, P10 tip size, Burlington, MA, USA) according to the manufacturer's instructions and loaded onto in‐house packed capillary columns (20 cm length, 75 µm inner diameter, Dr. Maisch GmbH, Ammerbruch, Germany, RepsoSil pur C18 material with pore size 120 Å and particle size 1.9 µm) via an Easy nLC1000 LC (Thermo Fisher Scientific, Waltham, MA, USA) coupled to a Q Exactive mass spectrometer (Thermo Fisher Scientific, Waltham, MA, USA) in data‐dependent acquisition mode and separated using a non‐linear binary gradient (131 min) from 1% to 99% solvent B (99.9% ACN [v/v], 0.1% acetic acid [v/v]) in solvent A at a constant flow rate of 300 nL/min. The MS1 scan was recorded in the orbitrap with a resolution of 140,000 at 200 m/z and a mass window of 300 to 1650 m/z. The 15 most abundant precursor ions were selected for higher‐energy C‐Trap dissociation fragmentation with enabled dynamic exclusion.

MS/MS spectra were searched using a two‐step searching strategy [48]. First, all MS/MS spectra were searched against a sample‐specific, non‐redundant metagenome‐derived database as described in the Supporting Information using Mascot [49] (Table S2) with the following parameters: fragment ion mass tolerance and parent ion tolerance of 10 ppm, no missed cleavages, variable modification on methionine (oxidation), and fixed modification on cysteine (carbamidomethylation). Search outputs from replicates were combined in Scaffold [50] (using X!Tandem [51] for validation with default settings). Peptide and protein identifications were accepted with a probability higher than 95% and 99%, respectively. The eight subset databases for each time point (including only identified proteins) were combined, and after filtering for redundancy using CD‐HIT [52] (clustering threshold of 97% identity), decoy entries were added, and all MS/MS spectra were searched again with the constructed non‐redundant subset database (88,300 entries including reverse entries). The Mascot [49] outputs were analyzed in Scaffold [50] as described above. Peptide and protein identifications were accepted if they contained at least two unique peptides, whereas this criterion was not applied to the first searching step. Peptide probabilities from Mascot and X!Tandem were assigned by the Scaffold Local FDR algorithm or Peptide Prophet algorithm [53] with delta‐mass correction, respectively. Protein identifications were accepted if they could be established at greater than 99% probability by the Protein Prophet algorithm [54] and contained at least two unique peptides. Proteins that contained similar peptides and could not be differentiated based on the MS/MS analysis alone were grouped to satisfy the principles of parsimony. The mass spectrometry proteomics data have been deposited to the ProteomeXchange Consortium (https://proteomecentral.proteomexchange.org) via the PRIDE partner repository [55] with the dataset identifier PXD055396. Functional and taxonomic annotation was performed by an in‐house pipeline. For this aim, protein sequences were mapped onto 2020 high‐quality MAGs [42] using Diamond BLASTP (v2.1.1.155, flags: –evalue 1E‐4 –id 95 –query‐cover 70 –subject‐cover 70. Sequences that remained unmapped were classified as either “prokaryotic” or “non‐prokaryotic” using BASTA (v1.4.1, with the options: –m 1 –i 0 –l 0 –e 0.001 –p 90), along with Diamond BLASTP (options: –evalue 0.001 –k 100) against the NCBI non‐redundant protein database (“NCBI_nr”; as of 22nd February 2023). For “non‐prokaryotic” sequences, the NCBI_nr results were used for Last Common Ancestor predictions via BASTA, while “prokaryotic” sequences were classified based on Diamond BLASTP results against the Genome Taxonomy Database (“GTDB”; r214.1) [56] with identical thresholds.

All LC‐MS/MS measurements generated a total of 20.5 million recorded spectra, of which 5.9 million could be confidently identified. On average, 858,049 (± 132,309) spectra were generated, of which 245,663 (± 45,788) were identified per sample, resulting in an average identification rate of 28.8% (± 5.0%). In classical metaproteomic studies, spectral identification rates typically range from 5% to 30% [5]. A cross‐laboratory (Critical Assessment of MetaProteome Investigation—CAMPI) study on stool samples reported identification rates ranging from 15.7% to 41.9% (average 23.2%) [4]. This variation was attributed to differences in protein extraction, sample preparation, and mass detection, followed by the same search strategy between the different labs using the X!Tandem search engine and a publicly available database.

To check the quality and robustness of the data, we calculated the squared Pearson correlation coefficient (*R* ^2^) of the weighted spectral counts for protein groups as a measure of the linear correlation between two individual technical replicates (Figure 1). For each pairwise combination of the three replicates of each sampling time point, the *R* ^2^ values indicated a high reproducibility. All R^2^ values between replicates exceeded 0.94. These *R* ^2^ values are comparable to stool samples that were analyzed directly after collecting fecal specimens compared to the same sample after being frozen for 2 months [57]. Only samples from the 7th May at 9 pm were an exception with a lower correlation value of 0.73 between replicates 1 and 3.

**FIGURE 1:**
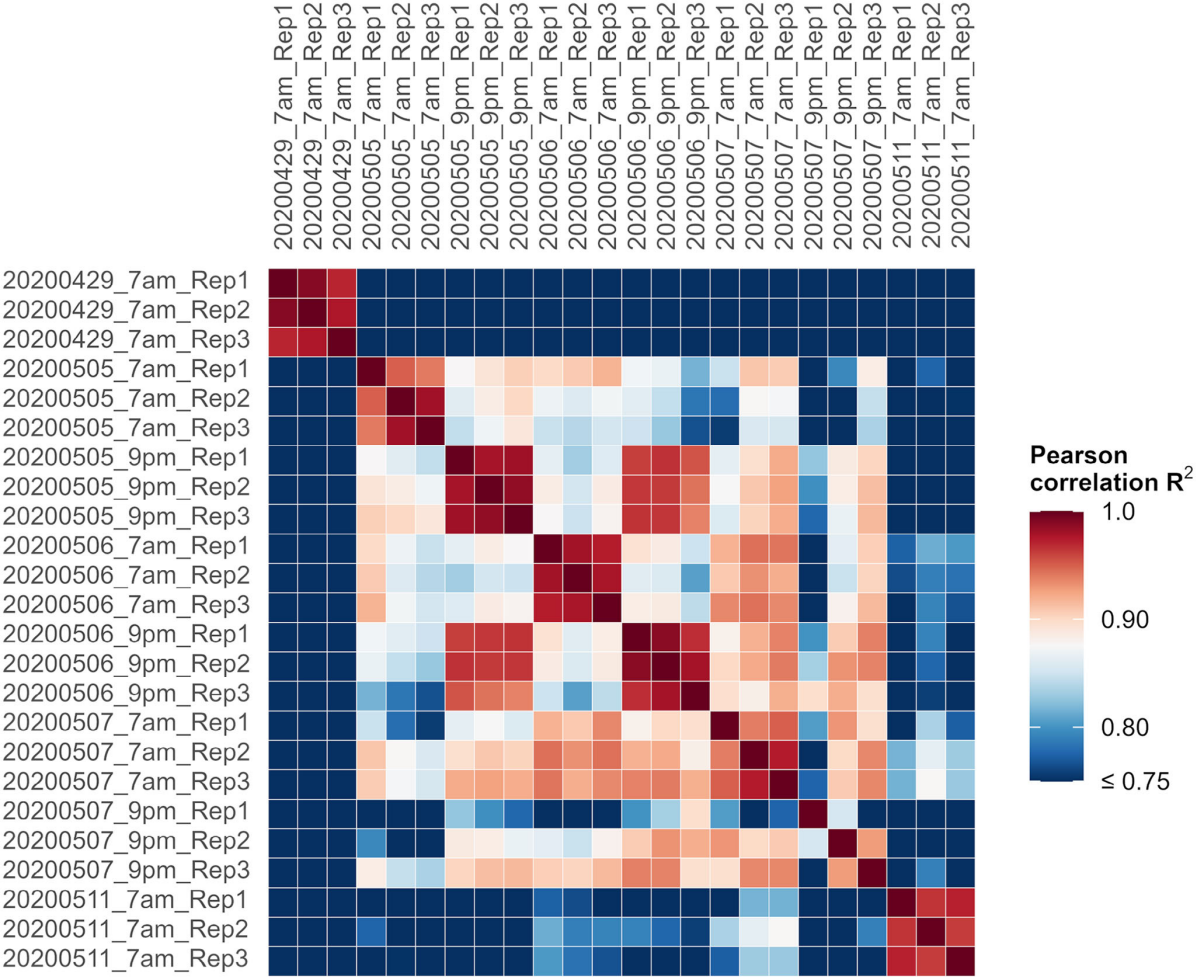
Heatmap representing the squared Pearson correlation coefficients (*R* ^2^) of the raw quantitative values (weighted spectra) from different samples taken between the 29th April and the 11th May 2020. The color gradient represents the correlation, with red indicating higher correlation values and blue representing lower correlation values. The x‐ and y‐axes correspond to the different samples.

In total, we identified 26,263 protein groups in the complete dataset (excluding contaminations and decoy hits) ranging from 6961 (7th May 2020, 9 pm, replicate 2) to 13,066 protein groups (6th May 2020, 7 am, replicate 1) per sample (with an average of 10,759 ± 1536) (Table S3). More spectra were identified per protein group in the 9 pm samples (considering weighted spectra, which represent the sum of unique peptide spectra per protein group and a fractional contribution of spectra from shared peptides, on average 24.8 identified spectra per protein group, with a 5% to 95% percentile of 1.86 to 88.5) compared to the 7 am samples (on average 21.0 identified spectra per protein group, with a 5% to 95% percentile of 1.81 to 76.7). After filtering for protein groups present in at least two of three replicates in at least one sampling time point, we quantified 19,933 protein groups, with numbers ranging from 7684 (7th May 2020, 9 pm) to 12,248 (6th May 2020, 7 am) protein groups per sampling time point. On average, significantly more protein groups were identified and quantified in the 7 am samples (11,426 ± 1250 identified and 11,110 ± 1032 quantified) compared to the 9 pm samples (9561 ± 1226 identified and 9273 ± 1145 quantified, paired Student's *t*‐test, *p* value < 0.0002).

At the end of April 2020, the first phase of the spring phytoplankton bloom that was dominated by *Ditylum brightwellii* diatoms ended, and a second bloom was dominated by *Cerataulina pelagica* and to a lesser extent *Chaetoceros* sp. diatoms [42]. At the same time, total bacterial cell counts increased again after a decline of *D. brightwellii* numbers towards the end of the first bloom. The frequency of metagenomic 16S rRNA gene sequences indicated a shift from *Alphaproteobacteria* dominating during the first blooming phase towards *Bacteroidota* taking over at the beginning of the second phytoplankton bloom [42]. Consequently, a noticeable shift in the metaproteome composition was detected over the full 13‐day period between 29th April and 11th May 2020. In total, 4379 protein groups in our dataset were quantified (in at least two of the three replicates) in all samples, representing the largest overlap (22.0%) compared to all possible intersections between the eight sampling time points (Figure S1). The first (29th April) and last (11th May) sampling time points differed from the others, with 2531 (12.7%) and 1426 (7.2%) protein groups being detected exclusively in these two sampling time points, respectively. Furthermore, 1048 (5.3%) protein groups were detected in all samples except on 29th April, confirming the overall shift in bacterioplankton composition in the second bloom phase. The semi‐diurnal shifts measured from 5th to 7th May were smaller than the shifts within 4 to 6 days.

To test for short‐term dynamics on the level of summed protein group abundances between the different taxonomic groups in our dataset, we initially compared alpha‐diversity based on the Shannon index calculated for the genus level between early and late samples from 5th to 7th May (Figure S2A). This metric describes the taxonomic richness and evenness of the abundance distribution of a microbial community within a sample with greater values indicating a more diverse community. A significantly higher diversity was detected in 7 am samples compared to 9 pm samples (paired Student's *t*‐test, *p* value < 0.0002).

In the spring bloom study of 2020, we investigated bacterioplankton metagenomes from 30 time points (including the eight time points we analyzed here) from the same bloom, which allowed us to reconstruct a total of 251 non‐redundant metagenome‐assembled genomes (MAGs) of free‐living bacteria [42]. We mapped the 19,933 protein groups quantified in our dataset back to these MAGs resulting in 11,622 (59%) protein groups mapping to 198 MAGs (79% of all MAGs detected). This indicates that the constructed MAGs represented the most abundant and active taxa and underscores the high quality of the metaproteome dataset. A phylogenetic tree was generated based on 16S rRNA sequences for individual MAGs, previously [42]. Using summed protein group abundances from those mapped protein groups, pair‐wise weighted Unifrac distances were calculated as a measure of beta diversity. These distances were then used as the input for a principal coordinate analysis (PCoA, Figure S2B). All sample points were separated from each other with days being sorted along the x‐axis and sampling time along the y‐axis.

After these general trends, we analyzed short‐ and long‐term trends across various taxonomic levels based on summed protein group abundances. The increase in protein group abundances of the bacterial community (Figure S3A) at the beginning of the *C. pelagica*‐dominated phytoplankton bloom phase could be mainly attributed to changes in the dominating phyla *Bacteroidota* and *Pseudomonadota* (formerly *Proteobacteria*) (Figure S3B). However, less abundant phyla, such as *Campylobacterota*, also exhibited long‐term changes in protein group abundances, whereas the phylum *Actinobacteriota* exhibited short‐term fluctuations between the 7 am and 9 pm samples. The dominant classes *Alphaproteobacteria*, *Gammaproteobacteria*, and *Bacteroidia* were subject to strong fluctuations (Figure 2), which mainly involved the orders *Rhodobacterales, Pelagibacterales*, *Pseudomonadales*, *Enterobacterales*, and *Flavobacteriales* (Figure S3D). *Rhodobacteraceae*, *Porticoccaceae*, *Pelagibacteraceae*, and *Alteromonadaceae* (Figure S3E) were dominating proteobacterial families including the abundant genera *Amylibacter*, *Planktomarina* and members of the gammaproteobacterial SAR92 (HTCC2207) clade (Figure S3F). Especially the dominant genera *Cd*. Prosiliicoccus (previously *Ulvibacter*) and *Aurantivirga* (SCGC‐AAA160‐P02) (Figure S3F) of the family *Flavobacteriaceae* showed higher abundance at the later sampling time points (Figure S3E). A rapid increase of *Bacteroidota* abundance after 29th April has already been shown for corresponding metagenomic 16S rRNA gene frequencies [42]. The indicated daytime‐dependent changes in abundances in the metagenomic gene frequencies were confirmed with our metaproteomic data, for the abundant genera *Amylibacter*, *Polaribacter*, and *Aurantivirga*.

**FIGURE 2:**
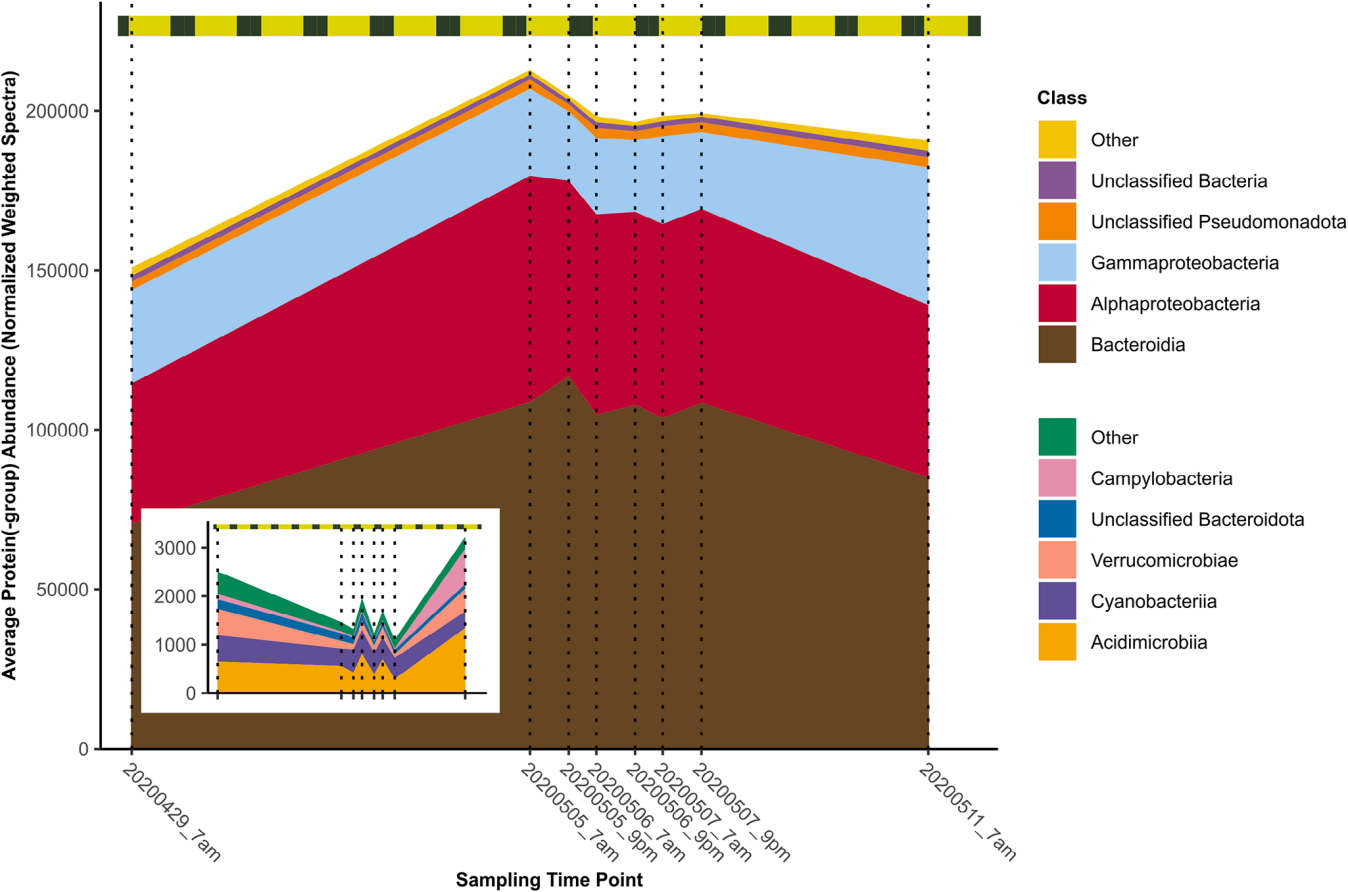
The composition of marine microbiota between the 29th April and 11th May 2020 at the Helgoland LTER station “Kabeltonne” in the North Sea based on metaproteomics data. The quantified protein groups assigned to the class level were summed. Low‐abundant members of taxonomical groups were merged to “Others”. The smaller inset shows additional classes that are included as “Others” in the main plot. In the absence of an assignment to any taxonomical group, respective proteins were assigned as unclassified to the next higher taxonomic level. The vertical dotted lines indicate the different sampling time points. The time between sunrise and sunset is indicated in yellow and the time between sunset and sunrise in black in the horizontal bar at the top of the plot (weather data, Table S1).

We identified significant changes in 65 relevant bacterial genera based on summed protein group abundances between early and late samples taken between 5th May and 7th May. As depicted in Figure 3, the different protein abundances between early and late samples of these genera were clustered depending on their abundance in all eight sampling time points (including 29th April and 11th May). The most interesting cluster consisted of genera with low overall protein group abundances at the beginning of May and higher protein group abundances in the 9 pm samples than the 7 am samples. This group consisted of the previously mentioned *Cd*. Prosiliicoccus and *Aurantivirga*, which were also more abundant at the beginning of the second bloom phase (peak on 6th May for *Cd*. Prosiliicoccus and 8th May for *Aurantivirga*) as indicated by the abundances inferred from metagenomic data or microscopic cell counting via catalyzed reporter deposition‐fluorescence in situ hybridization [42]. For most other clusters, protein groups were more abundant in the 7 am samples than the 9 pm samples, although these clusters showed different protein group abundances on the 29th April and 11th May. Members of the *Alteromonadaceae*, *Pelagibacteraceae*, and *Methylophilaceae* families showed the highest protein group abundance for the 5th and 6th May, whereas the cluster containing unclassified *Actinomycetota*, unclassified *Gammaproteobacteria* and *Poseidonibacter* showed highest abundance on 11th May and the cluster containing *Fabibacter* peaked at the end of the first phytoplankton bloom phase (29th April).

**FIGURE 3:**
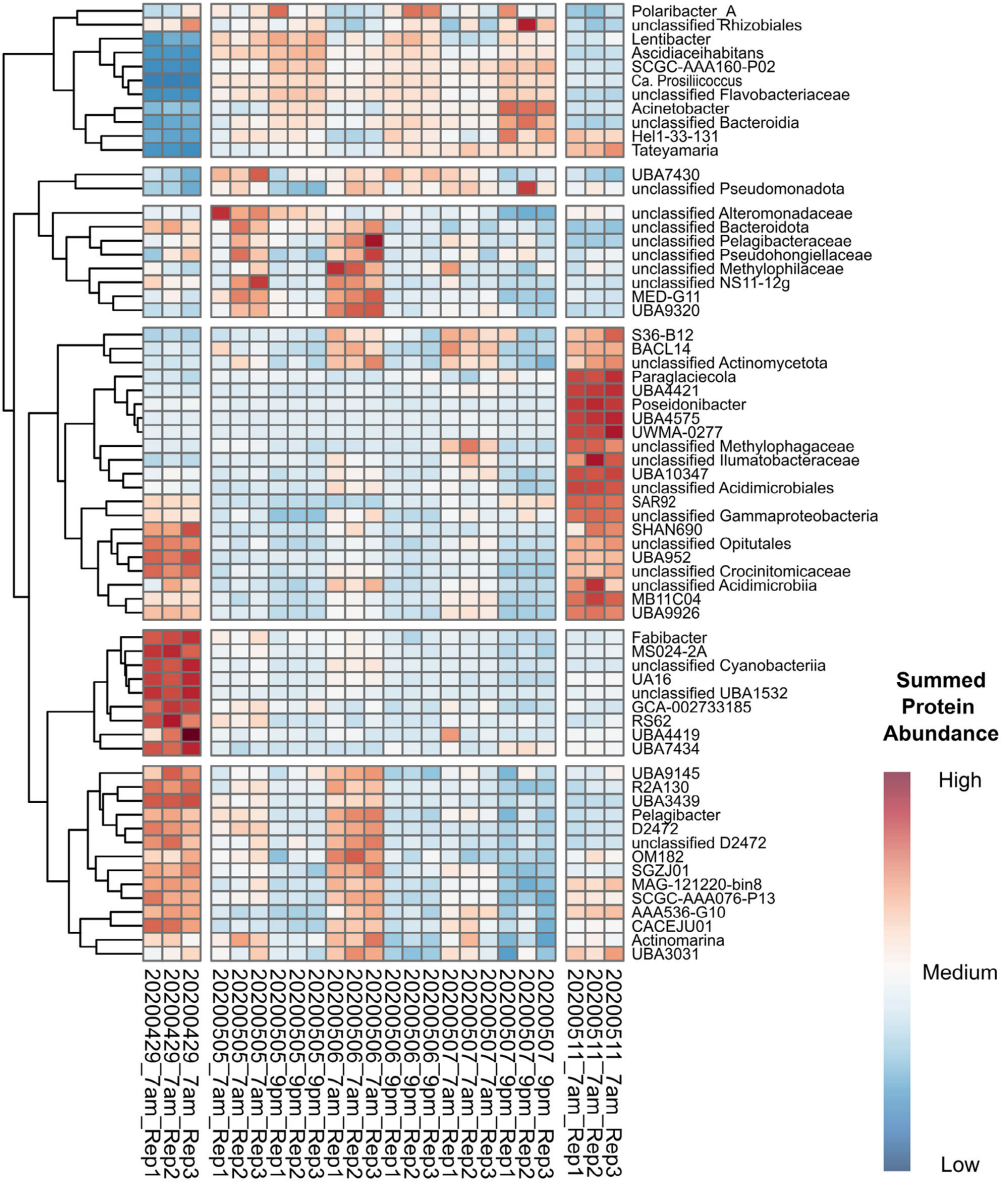
Heatmap and hierarchical clustering of significantly changed genera. The metaproteomic dataset from 5th May (7 am) until 7th May (9 pm) was analyzed using the R package ANCOMBC2 using summed protein abundances on the genus level. Significantly changed genera were hierarchically clustered and protein abundances of 29th April and 11th May were included.

Finally, we were interested in the function of the proteins with significant short‐term abundance changes. For this purpose, protein groups with significantly different abundances were identified using the R package DEqMS [58] (Table S3, labeled with “both” in the column DEqMS result). The analysis identified 422 such protein groups, 369 of which were more abundant in the morning samples. Functional annotation was performed using MAG annotations. For sequences that could not be assigned to MAGs, BLAST alignments of the protein sequences were conducted against the Genome Taxonomy Database (GTDB) [56] to find the most similar and related sequence. While 72 of the different abundant proteins were annotated as hypothetical proteins, the second highest number of differentially abundant proteins (47 significantly changed protein groups) were annotated as TonB‐dependent receptors, which mediate substrate‐specific transport across the outer membrane [59].

Previous studies have shown that bacterioplankton composition is highly dynamic during spring in the North Sea [20, 22, 43, 44, 60, 61, 62, 63, 64]. Changes over several days are more pronounced compared to the changes that occur within a day, as is shown in both our analysis and metagenome gene frequency analyses [42]. Nevertheless, our comprehensive metaproteome dataset suggests that significant changes occurred in the abundances of distinct bacterial protein groups at both, the taxonomic and functional levels, between the early and late samples. An even higher temporal resolution would be necessary for a more detailed analysis of diurnal adaptive changes of the bacterial physiology in the complex microbiomes during phytoplankton blooms. We anticipate that this study will pave the way towards such studies, and hope that the presented dataset will be a useful resource for researchers during the development of data evaluation and modeling approaches. Additionally, our preliminary study reveals that the timing of sampling is crucial in long‐term studies. In the case of irregular sampling, detected changes could be caused by diurnal changes and the associated changes in environmental conditions like the solar irradiation intensity. Changes at the transcriptome level are more dynamic as shown for dial oscillation in phototrophic bacteria [31]. Yet, as we show here, changes do also occur at the protein level. Owing to contemporary refined metaproteomics, such changes can nowadays be detected even in highly complex, diverse, and dynamic systems in situ.

## Conflicts of Interest

The authors declare no conflicts of interest.

## Supporting information




**Supporting Figure 1**: pmic70001‐sup‐0001‐figuresS1‐S3.docx

**Supporting Table 1**: pmic70001‐sup‐0001‐tableS1.xlsx

**Supporting Table 2**: pmic70001‐sup‐0001‐tableS2.xlsx

**Supporting Table 2**: pmic70001‐sup‐0001‐tableS3.xlsx

## Data Availability

The Mass Spectrometry Proteomics Data Have Been Deposited to the ProteomeXchange Consortium (https://proteomecentral.proteomexchange.org) via the PRIDE partner repository with the dataset identifier PXD055396.
