# Supplementary material for: Metaproteomic Dataset on Semi‐Diurnal Variability of the Bacterioplankton Communities During a Spring Phytoplankton Bloom in the North Sea
Source: Proteomics. 2025 Jun 23;25(17-18):19–27. doi: 10.1002/pmic.70001 (PMC12456260; doi:10.1002/pmic.70001)
Supplement: Supplementary file 1 — Supporting Figure 1: pmic70001‐sup‐0001‐figuresS1‐S3.docx [file PMIC-25--s004.docx]

**Supporting Information**

**Metaproteomic dataset on semi-diurnal variability of the bacterioplankton communities during a spring phytoplankton bloom in the North Sea**

Vaikhari Kale^1^, Jürgen Bartel^1^, Daniel Bartosik^2,3^, Philip Berhard Lude^1^, Chandni Sidhu^4^, Hanno Teeling^4^, Rudolf Amann^4^, Thomas Schweder^2,3^, Dörte Becher^1,3^, Anke Trautwein-Schult^1^

^1^ University of Greifswald, Institute of Microbiology, 17489 Greifswald, Germany

^2^ University of Greifswald, Institute of Pharmacy, 17489 Greifswald, Germany

^3^ Institute of Marine Biotechnology e.V., 17498 Greifswald, Germany

^4^ Max Planck Institute for Marine Microbiology, 28359 Bremen, Germany

Samples for comparing short-term proteome dynamics were collected as previously described at 1 m depth for three consecutive days at 7 am and 9 pm on 5^th^, 6^th^ and 7^th^ May 2020 ^[1]^. In addition, long-term proteome changes were analysed using samples collected six days before these samples (29^th^ April 2020, 7 am) and four days after these samples (11^th^ May 2020, 7 am). Sampled seawater was sequentially filtered through polycarbonate membrane filters (diameter 142 mm) with differing pore sizes (10 µm, 3 µm and 0.2 µm). The metaproteomic analysis of the 0.2 µm filter has been previously described in detail ^[1]^. For the replicates, one-eighth of the same filter was used. Although these are technical replicates, the replicates can also be considered biological replicates due to their non-homogeneous sample composition. Proteins were extracted from one-eighth of a 0.2 µm filter by adding lysis buffer, heating and sonication treatment. After centrifugation (1 min, 4 °C, 5,000 x g), the supernatant was used for protein precipitation by adding pre-cooled trichloroacetic acid (20% (v/v)). The precipitate was pelleted via centrifugation (1 h, 4 °C, 12,000 x g), washed three times with pre-cooled acetone and dried. The remaining protein pellet was resuspended in 2x Laemmli SDS sample loading buffer (4% SDS, 20% glycerol, 10% 2-mercaptoethanol, 0.002% bromophenol blue in 0.125 M Tris-HCl (pH 6.8)) and incubated for 5 min at 95 °C before separation via SDS-PAGE (Criterion TG 4–20% Precast Midi Gel, BIO-RAD Laboratories, Inc., Hercules, CA, USA). Afterwards, the gel was fixated (40% ethanol (v/v), 10% acetic acid (v/v)), and stained with Coomassie, before each gel line was cut into 20 equally sized pieces. According to Bonn *et al.*, the gel pieces were destained three times with washing buffer (200 mM ammonium bicarbonate in 30% ACN (v/v)) and dehydrated before the proteins were in-gel reduced and alkylated ^[2]^. The supernatant was discarded and after washing, dehydrating and drying, the gel pieces were covered with 120 µL trypsin solution (2 µg/mL trypsin, Promega, Fitchburg, WI, USA). After 20 min, the remaining supernatant was discarded and the gel pieces were incubated for 15 h at 37 °C. Peptides were eluted first with 120 µL solvent A (0.1% acetic acid (v/v)) for 15 min in an ultrasonication bath (SONOREX SUPER RK 102 H, Bandelin, Berlin, Germany) and second with 100 µL 30% ACN (v/v) for 15 min. The eluates were pooled and concentrated in a vacuum centrifuge before the peptides were desalted via ZipTip C18 (Merck Millipore, P10 tip size, Burlington, MA, USA) according to the manufacturer’s instructions. The peptides were dried in a vacuum centrifuge and resuspended in 10 µL 0.5x iRT standards kit (Biognosys, Schlieren, Swiss) in solvent A. Peptides were loaded onto in-house packed capillary columns (20 cm length, 75 µm inner diameter, Dr. Maisch GmbH, Ammerbruch, Germany, RepsoSil pur C18 material with pore size 120 Å and particle size 1.9 µm) via Easy nLC1000 LC (Thermo Fisher Scientific, Waltham, MA, USA) coupled to a Q Exactive mass spectrometer (Thermo Fisher Scientific, Waltham, MA, USA) in data-dependent acquisition mode and separated using a non-linear binary gradient (131 min) from 1% to 99% solvent B (99.9% ACN (v/v), 0.1% acetic acid (v/v)) in solvent A at a constant flow rate of 300 nL/min. The MS1 scan was recorded in the orbitrap with a resolution of 140,000 at 200 m/z and a mass window of 300 to 1,650 m/z. The 15 most abundant precursor ions were selected for higher-energy C-Trap dissociation fragmentation with enabled dynamic exclusion.

Metagenome sequencing was performed in long-read HiFi mode on a PacBio Sequel II (Pacific Biosciences, Menlo Park, CA, USA) using an SMRT cell per sample. Details on the raw data are provided by Sidhu *et al.* ^[3]^; assemblies were generated using Flye (version 2.8.3 ^[4]^) with the *-meta* and *-pacbio-hifi* options, followed by ORF calling with Prodigal (version 2.6.3 ^[5]^). MS/MS spectra were searched using a two-step searching strategy as previously described by Jagtap *et al.* ^[6]^. For the first searching step, all MS/MS spectra from the three replicates of one-time point were searched against a sample-specific, non-redundant metagenome-derived database (containing forward entries and common lab contaminations; redundant entries were removed via Seqkit ^[7]^) using Mascot (version 2.7.0.1) ^[8]^ with the following parameters: fragment ion mass tolerance and parent ion tolerance of 10 ppm, no missed cleavages, variable modification on methionine (oxidation), and fixed modification on cysteine (carbamidomethylation). Scaffold (version 5.0.1) ^[9]^ was used to merge the search results and validate MS/MS-based identifications of the three replicates per sample. During data analysis in Scaffold, an additional X!Tandem search was performed for validation (version 2017.2.1.4; The GPM, thegpm.org; version X!Tandem Alanine) ^[10]^ with default settings. Peptide and protein identifications were accepted if they could be established at greater than 95% and 99% probability, respectively, containing at least one unique peptide. For each sampling time point, we constructed a smaller subset sequence database containing all proteins inferred from peptide spectrum matches in the first search. The eight smaller subset databases were combined, filtered for redundancy using CD-HIT ^[11]^ with a clustering threshold of 97% identity and completed by the addition of decoy entries. For the second search step, all MS/MS spectra were searched against the constructed non-redundant subset database (88,300 entries including reverse entries) using Mascot as previously described. Afterwards, Scaffold (including the additional X!Tandem search) was used to merge the search results and validate MS/MS-based identifications. Peptide identifications were accepted if they could be established at greater than 95% probability. Peptide probabilities from Mascot and X!Tandem were assigned by the Scaffold Local FDR algorithm or Peptide Prophet algorithm ^[12]^ with delta-mass correction, respectively. Protein identifications were accepted if they could be established at greater than 99% probability by the Protein Prophet algorithm ^[13]^ and contained at least two unique peptides. The application of a local FDR filtering approach is more conservative in minimising false-positive identifications compared to the widely used global FDR approach ^[14]^. Proteins that contained similar peptides and could not be differentiated based on the MS/MS analysis alone were grouped to satisfy the principles of parsimony. The mass spectrometry proteomics data have been deposited to the ProteomeXchange Consortium (http:/proteomecentral.proteomexchange.org) via the PRIDE partner repository ^[15]^ with the dataset identifier PXD055396. A precise allocation between the MS *.raw files, the databases and the individual search results is shown in **Supporting Information Table S2**.

For data analysis, the “normalised weighted spectra” from Scaffold software were used for further analysis. All plots and data analysis were generated in R (version 4.4.1) with ggplot2 (version 3.5.1), reshape2 (version 1.4.4), UpSetR (version 1.4.0), cowplot (version 1.1.3), mia (version 1.12.0), pheatmap (version 1.0.12), and ggtree (version 3.12.0). NSAF values were calculated, and analysed for significant changes on the taxonomical level with the R package ANCOMBC (version 2.6.0) and on the protein group level with DEqMS ^[16]^ (version 1.22.0).

Protein sequences were mapped onto 2020 high-quality MAGs ^[3]^ using Diamond BLASTP (v2.1.1.155, flags: --evalue 1E-4 --id 95 --query-cover 70 --subject-cover 70. Sequences that remained unmapped were classified as either 'prokaryotic' or 'non-prokaryotic' using BASTA (v1.4.1, with the options: -m 1 -i 0 -l 0 -e 0.001 -p 90), along with Diamond BLASTP (options: --evalue 0.001 -k 100) against the NCBI non-redundant protein database ('NCBI_nr'; as of February 22, 2023). For 'non-prokaryotic' sequences, the NCBI_nr results were used for Last Common Ancestor (LCA) predictions via BASTA, while 'prokaryotic' sequences were classified based on Diamond BLASTP results against the Genome Taxonomy Database ('GTDB'; r214.1) ^[17]^ with identical thresholds.

Wind direction data and cloud coverage (**Supporting Information, Table S1**) were obtained from the Climate Data Store of the Copernicus Climate Change Service ^[18]^. Physicochemical parameters, such as mean air temperature, sunshine duration, and precipitation were obtained from ^[19]^, while information regarding the sunrise, sunset, and day length was obtained from ^[20]^, and additional data concerning the tide were obtained from ^[21]^ (**Supporting Information, Table S1**).

**References:**

[1] Beidler, I., Steinke, N., Schulze, T., Sidhu, C., Bartosik, D., Zühlke, M.-K., . . . Schweder, T. (2024). Alpha-glucans from bacterial necromass indicate an intra-population loop within the marine carbon cycle. *Nature Communications, 15*(1), 4048. doi: 10.1038/s41467-024-48301-5

[2] Bonn, F., Bartel, J., Büttner, K., Hecker, M., Otto, A., & Becher, D. (2014). Picking vanished proteins from the void: how to collect and ship/share extremely dilute proteins in a reproducible and highly efficient manner. *Analytical Chemistry, 86*(15), 7421-7427. doi: 10.1021/ac501189j

[3] Sidhu, C., Kirstein, I. V., Meunier, C. L., Rick, J., Fofonova, V., Wiltshire, K. H., . . . Teeling, H. (2023). Dissolved storage glycans shaped the community composition of abundant bacterioplankton clades during a North Sea spring phytoplankton bloom. *Microbiome, 11*(1), 77. doi: 10.1186/s40168-023-01517-x

[4] Kolmogorov, M., Yuan, J., Lin, Y., & Pevzner, P. A. (2019). Assembly of long, error-prone reads using repeat graphs. *Nature Biotechnology, 37*(5), 540-546. doi: 10.1038/s41587-019-0072-8

[5] Hyatt, D., Chen, G.-L., LoCascio, P. F., Land, M. L., Larimer, F. W., & Hauser, L. J. (2010). Prodigal: prokaryotic gene recognition and translation initiation site identification. *BMC Bioinformatics, 11*, 119. doi: 10.1186/1471-2105-11-119

[6] Jagtap, P., Goslinga, J., Kooren, J. A., McGowan, T., Wroblewski, M. S., Seymour, S. L., & Griffin, T. J. (2013). A two-step database search method improves sensitivity in peptide sequence matches for metaproteomics and proteogenomics studies. *Proteomics, 13*(8), 1352-1357. doi: 10.1002/pmic.201200352

[7] Shen, W., Le, S., Li, Y., & Hu, F. (2016). SeqKit: A Cross-Platform and Ultrafast Toolkit for FASTA/Q File Manipulation. *PLoS One, 11*(10), e0163962. doi: 10.1371/journal.pone.0163962

[8] Perkins, D. N., Pappin, D. J. C., Creasy, D. M., & Cottrell, J. S. (1999). Probability-based protein identification by searching sequence databases using mass spectrometry data. *Electrophoresis, 20*(18), 3551-3567. doi: 10.1002/(sici)1522-2683(19991201)20:18<3551::Aid-elps3551>3.0.Co;2-2

[9] Searle, B. C. (2010). Scaffold: A bioinformatic tool for validating MS/MS-based proteomic studies. *Proteomics, 10*(6), 1265-1269. doi: 10.1002/pmic.200900437

[10] Craig, R., & Beavis, R. C. (2004). TANDEM: matching proteins with tandem mass spectra. *Bioinformatics, 20*(9), 1466-1467. doi: 10.1093/bioinformatics/bth092

[11] Li, W., & Godzik, A. (2006). Cd-hit: a fast program for clustering and comparing large sets of protein or nucleotide sequences. *Bioinformatics, 22*(13), 1658-1659. doi: 10.1093/bioinformatics/btl158

[12] Keller, A., Nesvizhskii, A. I., Kolker, E., & Aebersold, R. (2002). Empirical statistical model to estimate the accuracy of peptide identifications made by MS/MS and database search. *Analytical Chemistry, 74*, 5383-5392. doi: 10.1021/ac025747h

[13] Nesvizhskii, A. I., Keller, A., Kolker, E., & Aebersold, R. (2003). A statistical model for identifying proteins by tandem mass spectrometry. *Analytical Chemistry, 75*, 4646-4658. doi: 10.1021/ac0341261

[14] Käll, L., Storey, J. D., MacCoss, M. J., & Noble, W. S. (2008). Posterior error probabilities and false discovery rates: two sides of the same coin. *Journal of Proteome Research, 7*(1), 40-44. doi: 10.1021/pr700739d

[15] Perez-Riverol, Y., Bai, J., Bandla, C., García-Seisdedos, D., Hewapathirana, S., Kamatchinathan, S., . . . Vízcaino, J. A. (2022). The PRIDE database resources in 2022: a hub for mass spectrometry-based proteomics evidences. *Nucleic acids Research, 50*(D1), D543-D552. doi: 10.1093/nar/gkab1038

[16] Zhu, Y., Orre, L. M., Zhou Tran, Y., Mermelekas, G., Johansson, H. J., Malyutina, A., . . . Lehtio, J. (2020). DEqMS: A Method for Accurate Variance Estimation in Differential Protein Expression Analysis. *Mol Cell Proteomics, 19*(6), 1047-1057. doi: 10.1074/mcp.TIR119.001646

[17] Parks, D., & Hugenholtz, P. (2024). *Genome Taxonomy Database r214.1. Version 1.92.*

[18] Hersbach, H., Bell, B., Berrisford, P., Biavati, G., Horányi, A., Muñoz‐Sabater, J., . . . Thépaut, J. N. (2023). *ERA5 hourly data on single levels from 1940 to present. Copernicus Climate Change Service (C3S) Climate Data Store (CDS)*.

[19] Wetterkontor.de. Retrieved 17.09.2024, from <https://www.wetterkontor.de/de/wetter/deutschland/rueckblick.asp?id=84&datum0=16.04.2020&datum1=29.04.2020&jr=2024&mo=9&datum=11.05.2020&t=2&part=2>

[20] Sonnenzeiten.org. Retrieved 17.09.2024, from <https://sonnenzeiten.org/suchen?location=Helgoland,%20Germany&year=2020&month=4#calendar>

[21] Gezeitenfisch.com. Retrieved 17.09.2024, from <https://gezeitenfisch.com/de/schleswig-holstein/helgoland-binnenhafen>

**Supporting Information**

**Figure legends:**


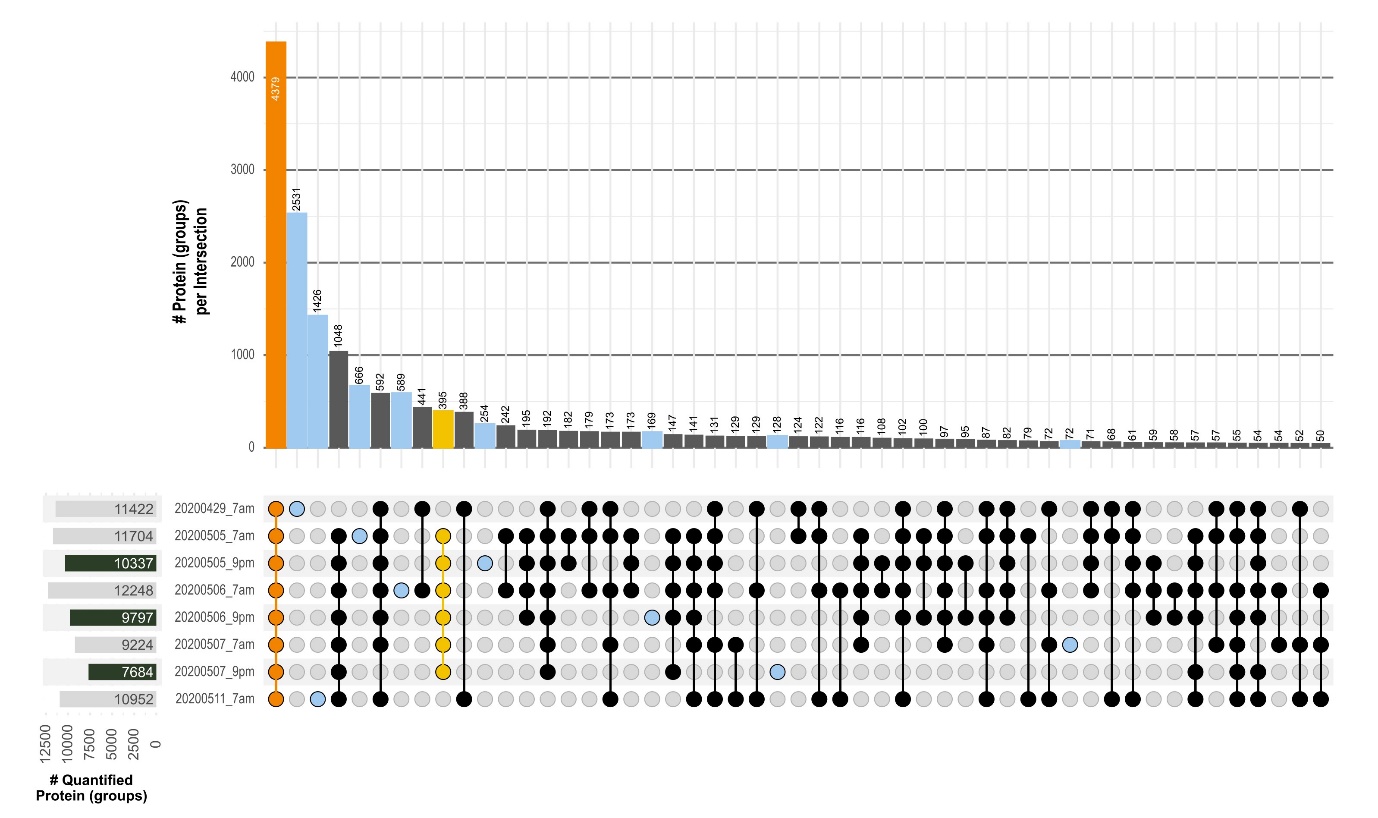


**Figure S1: UpSet plot illustrating the number of unique and shared protein groups quantified in the dataset. Intersections represented by less than 50 protein groups were excluded. Each bar represents the number of protein groups quantified in specific combinations of sampling time points. The coloured bars represent distinct groups of samples: orange highlights the intersection of proteins detected in all samples, yellow indicates proteins quantified between 5^th^ May (7 am) and 7^th^ May (9 pm), and blue represents the unique protein groups from each sampling time point. In the left, lower corner, the number of quantified protein groups for each sampling time point are shown.**


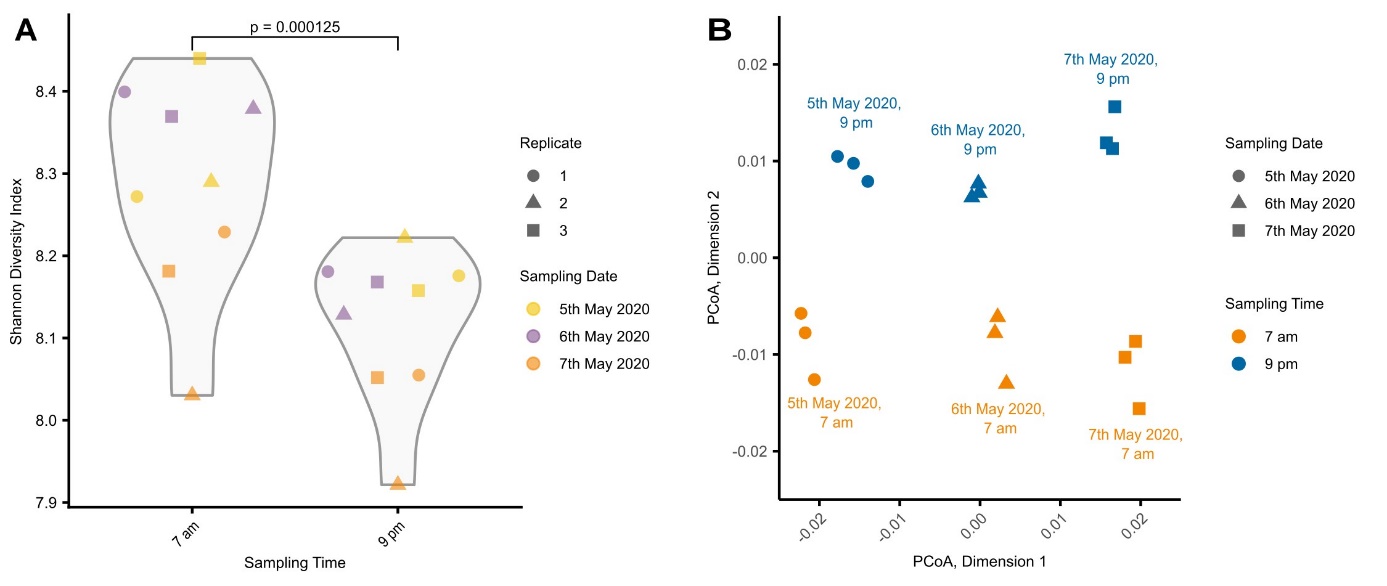


**Figure S2: A Violin plot of Shannon alpha-diversity index calculated for the genus level for the 7 am and the 9 pm samples from 5^th^ to 7^th^ May 2020. The paired Student’s t-test indicates a statistical significance of the differences. B Principal coordinated analysis of the Beta-diversity calculated from pair-wise protein group abundance weighted Unifrac distances. The sampling date and time are indicated as text near each sample cluster.**


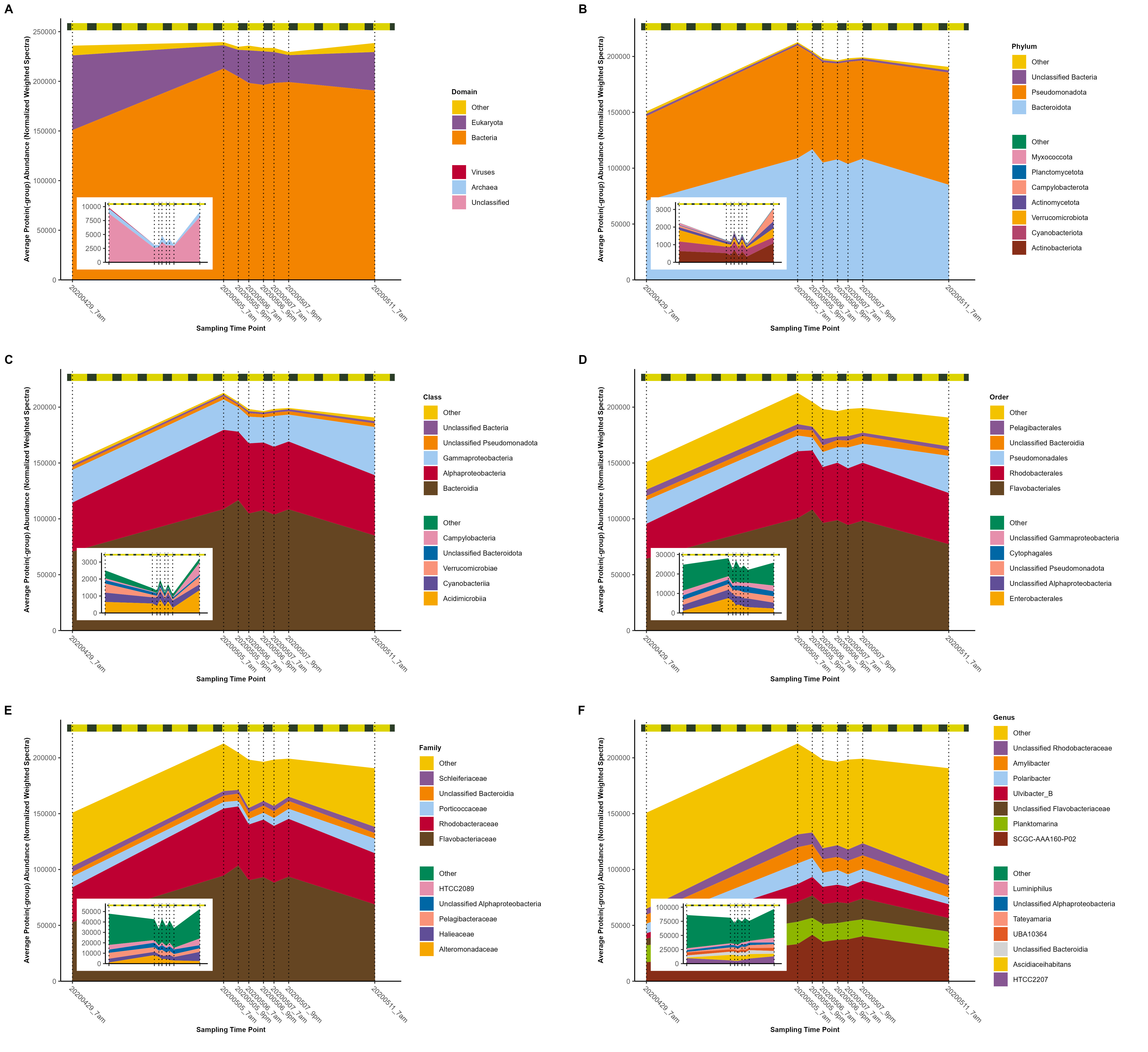


**Figure S3: The** composition of marine microbiota during the 29^th^ April and 11^th^ May 2020 at the Helgoland LTER station “Kabeltonne” in the North Sea based on metaproteomics data. The quantified protein groups assigned to the taxonomical level **A** domain, **B** phyla, **C** class, **D** order, **E** family, and **F** genus were summed. Low abundant members of taxonomical groups were merged to ‘Others’. The smaller insets show additional members of each taxonomical group that are included as ‘Others’ in the main plot. In absence of an assignment to any taxonomical group, respective proteins were assigned as unclassified to the next higher taxonomic level. The vertical dotted lines indicate the different sampling time points. The time between sunrise and sunset is indicated in yellow and the time between sunset and sunrise in black in the horizontal bar on the top of the plot (weather data **Supporting Information, Table S1**).

**Table S1:**

**Sheet 1:** List of daily measured physicochemical parameters, such as mean air temperature, sunshine duration, precipitation, sunrise, sunset, and day length, and additional data concerning the tide.

**Sheet 2:** List of hourly measured wind direction data and mean wave direction were obtained from the Climate Data Store of the Copernicus Climate Change Service.

**Table S2:** List of all MS *.raw files, used databases (*.fasta file) and corresponding results file.

**Sheet 1:** List of all MS *.raw files, used databases (*.fasta file) and corresponding results file for the first searching step.

**Sheet 2:** List of all MS *.raw files, used databases (*.fasta file) and corresponding results file for the second searching step.

**Table S3:** List of identified and quantified protein groups. Laboratory contaminants and decoy hits were removed, and protein groups are listed by the accession number of the main protein.
